# Overexpression of UTX promotes tumor progression in Oral tongue squamous cell carcinoma patients receiving surgical resection: a case control study

**DOI:** 10.1186/s12885-021-08726-3

**Published:** 2021-09-01

**Authors:** Yen-Hao Chen, Chang-Han Chen, Chih-Yen Chien, Yan-Ye Su, Sheng-Dean Luo, Shau-Hsuan Li

**Affiliations:** 1 Department of Hematology-Oncology, Kaohsiung Chang Gung Memorial Hospital and Chang Gung University College of Medicine, No.123, Dapi Rd., Niaosong Dist, Kaohsiung City, 833 Taiwan; 2 School of Medicine, Chung Shan Medical University, Taichung, 402 Taiwan; 3 Department of Nursing, Meiho University, Pingtung, 912 Taiwan; 4 Institute of Medicine, Chung Shan Medical University, Department of Medical Research, Chung Shan Medical University Hospital, Taichung, 402 Taiwan; 5 Department of Otolaryngology, Kaohsiung Chang Gung Memorial Hospital and Chang Gung University College of Medicine, Kaohsiung, Taiwan

**Keywords:** UTX, Tongue cancer, Squamous cell carcinoma, Surgery

## Abstract

**Background:**

Ubiquitously transcribed tetratricopeptide repeat on chromosome X (UTX) has been identified as a histone 3 lysine 27 (H3K27) demethylase and acted as a tumor suppressor gene or oncogenic function. The current study was to explore the significance of UTX in oral tongue squamous cell carcinoma (OTSCC) patients who received surgical resection.

**Methods:**

A total of 148 OTSCC patients who underwent surgical resection were identified, including 64 patients (43%) with overexpression of UTX and 84 patients (57%) harboring low expression of UTX. We also used two OTSCC cell lines, SAS and Cal 27, to determine the modulation of cancer. Chi-square test was used to investigate the difference of categorical variables between the groups; survival outcome was analyzed using the Kaplan–Meier method in univariate analysis, and a Cox regression model was performed for multivariate analyses.

**Results:**

Univariate and multivariate analyses showed overexpression of UTX were significantly related to worse disease-free survival (*P* = 0.028) and overall survival (*P* = 0.029). The two OTSCC cell lines were treated with GSK-J4, a potent inhibitor of UTX, and transwell migration and invasion assays showed an inhibitory effect with a dose-dependent manner. In addition, western blot analyses also revealed the inhibition of cell cycle and epithelial-mesenchymal transition.

**Conclusion:**

Our study suggests that UTX plays an important role in the process of OTSCC and overexpression of UTX may predict poor prognosis in OTSCC patients who received surgical resection.

**Supplementary Information:**

The online version contains supplementary material available at 10.1186/s12885-021-08726-3.

## Background

Oral cavity cancer is one of the most aggressive cancers worldwide. The tongue is the most commonly occurring site for oral cavity cancers; such cancers are usually squamous cell carcinomas. Oral tongue squamous cell carcinoma (OTSCC) has the highest incidence, and while the incidence generally has increased, the five-year survival rates have not improved over the last 20 years [1, 2]. OTSCC usually migrates rapidly to the adjacent structures and disseminates to other organs through the bloodstream and lymphatic drainage. Surgical resection remains the gold standard for operable disease, and adjuvant chemotherapy, radiotherapy, or combination therapy is indicated for high-risk populations, in clinical practice [3]. The important risk factors of poor prognosis in OTSCC include depth of invasion, perineural invasion, lymphovascular invasion, increasing pathological T and N stage, extracapsular extension, and surgical margin [4–8]. These risk factors lead to an increasing incidence of locoregional failure and distant metastasis, contributing to poor prognosis and impaired quality of life [9, 10]. Thus, it is of great significance to identify a potential biomarker associated with tumor progression to improve prognosis in OTSCC patients.

Ubiquitously transcribed tetratricopeptide repeat on chromosome X (UTX)—also known as KDM6A—is a histone demethylase that targets di- and tri-methylated histone H3 lysine 27 (H3K27); it is involved in embryonic development, tissue-specific differentiation, and cancer growth [11, 12]. Growing evidence has shown that UTX mutations or deregulation are associated with several cancer types, including breast cancer, bladder cancer, colon cancer, and B-cell lymphoma [13–17]. However, the role of UTX in tumor suppression or in the enhancement of cancer cell proliferation still remains controversial. In breast cancer, blocking UTX resulted in a significant decrease in tumor cell proliferation and invasion in cell lines and in a mouse xenograft model. In addition, breast cancer patients showing overexpression of UTX were reported to have poor prognosis [13]. On the other hand, UTX is often associated with somatic loss-of-function mutations in several cancer types such as renal carcinoma, acute leukemia, medulloblastoma, etc. [18]. In addition, UTX transcriptionally activates Retinoblastoma (Rb) genes to inhibit tumor cell proliferation in several tumor types, suggestion that UTX is a tumor suppressor [19].

However, the role of UTX in OTSCC still remains unclear. We suppose that UTX overexpression is a novel mechanism contributing to the promotion of tumor cell proliferation in OTSCC patients. The aim of the current study was to investigate the role of UTX in the prognosis of OTSCC patients who underwent surgical resection.

## Methods

### Patient selection

Between January 2006 and December 2015, 1059 patients who were diagnosed with OTSCC at Kaohsiung Chang Gung Memorial Hospital were retrospectively reviewed. Patients with a history of a second primary malignancy or a distant metastasis—whether before or after the diagnosis of OTSCC—were excluded. Subsequently, those who received neoadjuvant treatment such as chemotherapy, radiotherapy, or combination therapy were also excluded, and patients who underwent curative surgical resection were selected. Among these, only patients with available paraffin embedded tissue blocks were enrolled. Finally, a total of 148 OTSCC patients were identified.

The Eastern Cooperative Oncology Group (ECOG) Scale of Performance Status (PS) is one such measurement and describes a patient’s level of functioning in terms of their ability to care for themselves, daily activity, and physical ability. All patients in our study were ECOG PS 0 or 1; PS 0 means normal activity and PS 1 means some symptoms, but still near fully ambulatory.

### Immunohistochemistry

In our study, all patients received glossectomy. The tissue blocks from the formalin-fixed paraffin wax-embedded OTSCC tissue (from glossectomy sample, not from biopsy sample) were cut to prepare sections with 4-μm thickness. For each patient, all slides from the glossectomy sample were carefully reviewing by two pathologists (WT Huang and SL Wang) and one slide which is the most significant for the OTSCC was selected for further investigation. First, the sections were subjected to deparaffinization by incubating them in a dry oven at 60 °C for 1 h, antigen retrieval using 10 mM citrate buffer (pH 6.0), followed by incubation in a hot water bath (95 °C) for 20 min, and peroxidase blocking using 0.3% hydrogen peroxide for 5 min. Then, a primary antibody against UTX (ab235989, 1:2000, Abcam, Cambridge, MA, USA) was added to the sections and allowed to react; subsequently, a ready-to-use visualization reagent consisting of a goat secondary antibody was added to the sections and was allowed to react. The tissue sections were then incubated with a polymer for 8 min, followed by staining with 3,3′-diaminobenzidine for 10 min, and counterstaining with hematoxylin. The negative control group samples were stained using an identical procedure, while a slide of hepatocellular carcinoma cells was used as a positive control. The slides were scored by two pathologists (WT Huang and SL Wang) who were blinded to the clinicopathological features or prognosis. The method used for scoring the expression of UTX was determined according to a previous published study [20].

The proportions of UTX-expressing tumor cells were scored using the immunoreactive score (IRS) system which is calculated by the product of the multiplying the staining intensity (0: none; 1: weak; 2: moderate; and 3: strong) and the percentage of positively stained cells (0: no staining; 1: < 10% of the cells; 2: 11–50%; 3: 51–80%; and 4: > 81%), resulting in IRS scores between 0 (no staining) and 12 (maximum staining) [21]. A specimen with a sum score of > 6 was regarded as having positive staining.

### Cell lines and culture

The OTSCC cell lines—SAS and Cal 27—were purchased from American Type Culture Collection and cultured in Dulbecco’s modified Eagle’s medium-nutrient mixture F-12 (Sigma–Aldrich). All culture media contained 10% fetal bovine serum. The cells were then cultured at 37 °C.

### Migration and invasion assays

Transwell inserts (pore size 8 mm; Corning, Glendale, AZ, USA) were used to evaluate cell migration, and Matrigel (BD Biosciences, San Jose, CA, USA) coated porous filters were used to examine cell invasion. Cells (1 × 10^4^) in 200 ml DMEM medium containing 10% FBS were seeded into inserts, and 600 ml was added in lower part of the well. Cells were incubated for 24 h. Cells on the upper side of the membrane were wiped, and cells moving to the other side of the filters were stained by crystal violet and counted using a microscope in three randomly selected fields. Independent experiments were repeated three times.

### Western blot analysis

Whole-cell lysates of GSK-J4-treated cells were extracted with 300 μL of RIPA buffer (50 mM Tris, 150 mM NaCl, 1% NP40, 0.5% sodium deoxycholate, and 0.1% sodium dodecyl sulfate [SDS]), and subjected to western blot analysis. The membranes were incubated with polyclonal antibodies against UTX (ab36938, 1:2000, Abcam, Cambridge, MA, USA), Tri-methylation of histone H3 lysine 27 (H3K27me3) (A-4039, 1:2000, EpiGentek, Farmingdale, New York, USA), CDK4 (#12790, 1:1000, cell signaling, Danvers, Massachusetts, USA), Cyclin D1 (#2978, 1:1000, cell signaling, Danvers, Massachusetts, USA), E-cadherin (GTX124178, 1:5000, Genetex, Irvine, CA, USA), N-cadherin (sc-7939, 1:500, Santa Cruz Biotechnology, Santa Cruz, California, USA), Twist1 (#46702S, 1:500, cell signaling, Danvers, Massachusetts, USA), and β-actin (A5441, 1:10000, Sigma-Aldrich, St. Louis, Missouri, USA). Horseradish peroxidase-conjugated anti-rabbit secondary antibody was added to detect primary antibodies, and blots were developed with a chemiluminescence system (Pierce). All resolved protein bands were developed using the Western Lightning Chemiluminescence Reagent Plus system (Amersham Biosciences). All the experiments were repeated at least three times with similar results.

### Ethics statement

Ethical approval for this study was obtained from the Chang Gung Medical Foundation Institutional Review Board (201901388B0). All procedures used in studies involving human participants were performed in accordance with the ethical standards of the institutional research committee and the World Medical Association Declaration of Helsinki. Written informed consent was waived by the Chang Gung Medical Foundation Institutional Review Board.

### Statistical analysis

Data of baseline characteristics were expressed as number and percentages appropriately. The chi-square test was used for comparing categorical variables. Disease-free survival (DFS) was defined as the time from surgery to recurrence of tumor or death from any cause without evidence of recurrence. Overall survival (OS) was calculated from the time of diagnosis of OTSCC to death or to the time of last living contact. Univariate analysis was performed using the Kaplan–Meier method, and differences were assessed with the log-rank test. The Cox proportional hazards model was used to identify independent prognostic factors in multivariate analysis. The statistical analysis was performed according to the protocol described in the previously published study [22].

We carried out all statistical analyses using SPSS software (International Business Machines Corp., New York, USA). A two-tailed *p* value of < 0.05 was considered to indicate statistical significance in all analyses.

## Results

### Patient characteristics

Between January 2006 and December 2015, a total of 148 OTSCC patients who received surgical resection at Kaohsiung Chang Gung Memorial Hospital were enrolled in the study. All of these patients had an Eastern Cooperative Oncology Group performance status ≤1. In our study, we enrolled 135 male patients and 13 female patients with a median age of 53 years (range: 26–86 years). A history of smoking was found in 122 patients (82%), alcohol consumption in 118 patients (80%), and betel-nut chewing was mentioned in 112 patients (76%). Forty-two patients (29%) had pathological T1 status, 48 patients (32%) had pathological T2 status, 12 patients (8%) had pathological T3 status, and 46 patients (31%) had pathological T4 status; the pathological N status data revealed 79 patients (54%) diagnosed as N0, 24 patients (16%) as N1, 42 patients (28%) as N2, and three patients (2%) as N3. There were 31 patients (21%) with stage I, 29 patients (20%) with stage II, 23 patients (15%) with stage III, 58 patients (39%) with stage IVA, and seven patients (5%) with stage IVB disease. The tumor grade data showed that 84 patients (57%) had grade 1, 59 patients (40%) had grade 2, and five patients (3%) had grade 3 tumors.

In our study, the median period of follow–up was 82.5 months (range: 60.7–110.6 months) for the 63 living survivors and 52.4 months (range: 1.0–110.6 months) for all 148 patients. The five-year DFS and OS rates were 44.6 and 47.3%, respectively. The relevant details are shown in Table 1.

**Table 1:** Characteristics of 148 patients with oral tongue squamous cell carcinoma receiving surgical resection

Age (years)	53 (range: 26–86)	
Sex		
	male	135 (91%)
	female	13 (9%)
Pathological T status		
	T1	42 (29%)
	T2	48 (32%)
	T3	12 (8%)
	T4a	41 (28%)
	T4b	5 (3%)
Pathological N status		
	N0	79 (54%)
	N1	24 (16%)
	N2	42 (28%)
	N3	3 (2%)
Pathological 8th AJCC Stage		
	I	31 (21%)
	II	29 (20%)
	III	23 (15%)
	IVA	58 (39%)
	IVB	7 (5%)
Histologic grade		
	1	84 (57%)
	2	59 (40%)
	3	5 (3%)
UTX expression		
	Overexpression	64 (43%)
	Low expression	84 (57%)
Vascular invasion		
	Absent	124 (84%)
	Present	24 (16%)
Perineural invasion		
	Absent	81 (55%)
	Present	67 (45%)
Extracapsular extension		
	Absent	111 (75%)
	Present	37 (25%)
Surgical margin		
	Negative	137 (93%)
	Positive	11 (7%)
Smoking		
	Absent	26 (18%)
	Present	122 (82%)
Alcohol		
	Absent	30 (20%)
	Present	118 (80%)
Betel-nut chewing		
	Absent	36 (24%)
	Present	112 (76%)

AJCC, American Joint Committee on Cancer

### UTX and clinical outcome

**Fig. 1:**
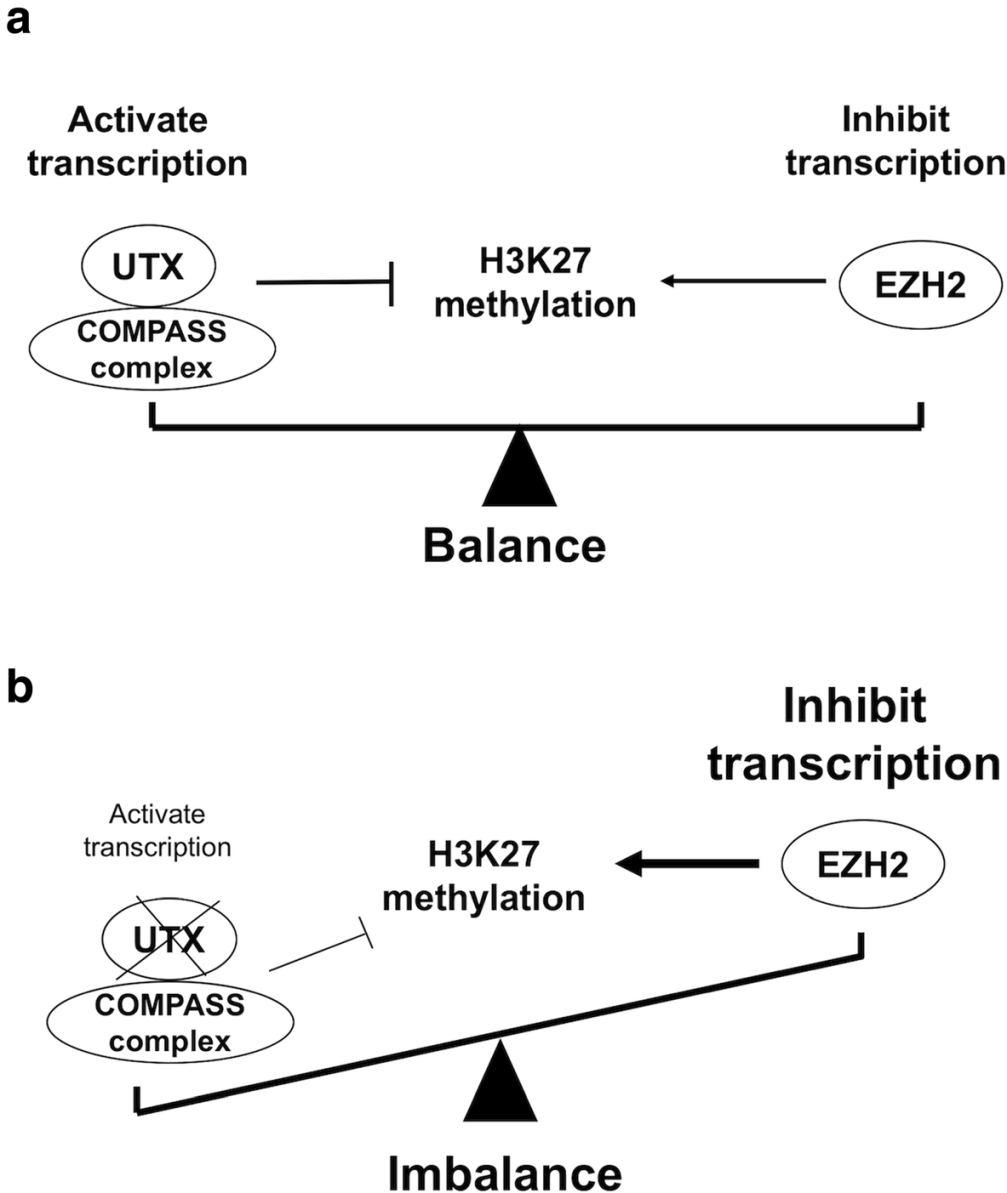
The mechanism of UTX in the tumor progression. (**a**) Balance of H3K27 methylation between UTX and EZH2 function in the tumor cell transcription; (**b**) Loss of UTX induces increased H3K27 methylation, leading to inhibition of tumor cell transcription

**Fig. 2:**
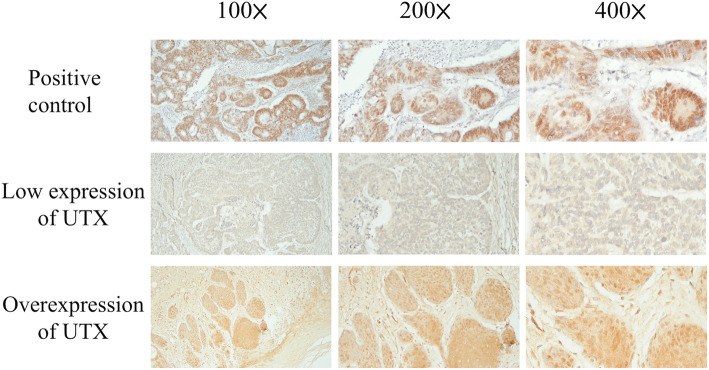
Results of the immunohistochemical analysis of UTX in oral tongue squamous cell carcinoma patients

The mechanism of UTX in the tumor cell progression was shown in Fig. 1. The expression of UTX in immunohistochemical staining is shown in Fig. 2. Among the 148 patients, there were 64 patients (43%) with overexpression of UTX, and 84 patients (57%) showing a low expression of UTX. N*o significant difference in baseline characteristics* including age, sex, pathological T status, histologic grade, vascular invasion, perineural invasion, surgical margin, cigarette smoking, alcohol consumption, and betel–nut chewing *was observed between groups categorized based on UTX expression levels*. Patients showing UTX overexpression had a higher percentage of pathological N status and tumor stage compared to those with low expression of UTX. The comparison is shown in Table 2.

**Table 2:** Associations between UTX expression and clinicopathological parameters in 148 patients with oral tongue squamous cell carcinoma receiving surgical resection

Parameters		UTX expression		
		Overexpression (*N* = 64)	Low expression (*N* = 84)	*P* value
Age				
	<53y/o	31 (48%)	39 (46%)	0.81
	≧53y/o	33 (52%)	45 (54%)	
Sex				
	male	58 (91%)	77 (92%)	0.82
	female	6 (9%)	7 (8%)	
Pathological T status				
	T1 + T2	34 (53%)	56 (67%)	0.10
	T3 + T4	30 (47%)	28 (33%)	
Pathological N status				
	N0	27 (42%)	52 (62%)	0.017*
	N1 + 2 + 3	37 (58%)	32 (38%)	
Pathological 8th AJCC Stage				
	I + II	19 (30%)	41 (49%)	0.019*
	III + IV	45 (70%)	43 (51%)	
Histologic grade				
	1	38 (59%)	46 (55%)	0.58
	2 + 3	26 (41%)	38 (45%)	
Vascular invasion				
	Absent	53 (83%)	71 (85%)	0.78
	Present	11 (17%)	13 (15%)	
Perineural invasion				
	Absent	31 (48%)	50 (60%)	0.18
	Present	33 (52%)	34 (40%)	
Extracapsular extension				
	Absent	43 (67%)	68 (81%)	0.06
	Present	21 (33%)	16 (19%)	
Surgical margin				
	Negative	58 (91%)	79 (94%)	0.43
	Positive	6 (9%)	5 (6%)	
Smoking history				
	Absent	12 (19%)	14 (17%)	0.74
	Present	52 (81%)	70 (83%)	
Alcohol history				
	Absent	11 (17%)	19 (23%)	0.42
	Present	53 (83%)	65 (77%)	
Betel-nut chewing history				
	Absent	11 (17%)	25 (30%)	0.08
	Present	53 (83%)	59 (70%)	

AJCC, American Joint Committee on Cancer. *Statistically significant

**Fig. 3:**
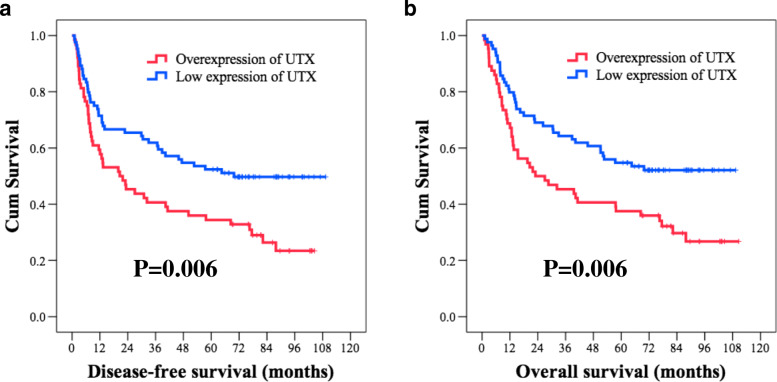
Comparison of Kaplan-Meier curves in oral tongue squamous cell carcinoma patients with high and low expression of UTX. (**a**) Disease-free survival (**b**) Overall survival

With respect to DFS, a univariate analysis found that age, sex, histologic grade, surgical margin, smoking, alcohol consumption, and betel–nut chewing were not statistically significant predictors of DFS. The 58 patients who had pathological T3–4 were found to have worse DFS than did the other 90 patients who had pathological T1–-2 disease (11.4 months versus 69.8 months, *P* = 0.004); 69 patients with nodal metastasis had shorter DFS in comparison with the other 79 patients without nodal metastasis (13.2 months versus 87.9 months, *P* < 0.001). Significantly inferior DFS was found in the 88 patients who had pathological stage III–IV than in the 60 patients who had pathological stage I–II disease (13.4 months versus not reached, *P* = 0.001). Patients who had positive vascular invasion, perineural invasion, and extracapsular extension were mentioned to have inferior DFS compared to those who did not (*P* = 0.018, *P* = 0.008, *P* < 0.001, respectively). The 64 patients who showed an overexpression of UTX had worse DFS than did the other 84 patients with a low expression of UTX (20.6 months versus 69.8 months, *P* = 0.006, Fig. 3A). In a multivariate analysis, extracapsular extension (P < 0.001, hazard ratio (HR): 2.22, 95% confidence interval (CI): 1.43–3.47) and overexpression of UTX (*P* = 0.028, HR: 1.61, 95% CI: 1.05–2.45) were independent prognostic parameters of worse DFS.

In the analysis of OS, no statistically significant differences in parameters such as age, sex, histologic grade, alcohol consumption, and betel–nut chewing were observed in univariate analysis. The 58 patients with pathological T3–4 were found to have inferior OS compared to the other 90 patients with pathological T1–2 disease (13.2 months versus 82.2 months, *P* < 0.001); 69 patients who had nodal metastasis had worse OS compared to the other 79 patients without nodal metastasis (14.8 months versus not reached, P < 0.001). Significantly inferior OS was observed in the 88 patients with pathological stage III–IV compared to that in the 60 patients with pathological stage I–II disease (22.5 months versus not reached, *P* < 0.001). Patients who had positive vascular invasion, perineural invasion, extracapsular extension, surgical margin, and smoking history were found to have shorter OS than those who did not (*P* = 0.004, *P* = 0.022, P < 0.001, *P* = 0.031, and P = 0.031, respectively). The 64 patients who showed an overexpression of UTX had worse OS than did the other 84 patients with a low expression of UTX (23.0 months versus 69.8 months, *P* = 0.006, Fig. 3B). Multivariate analysis showed that advanced pathological T status (*P* = 0.015, HR: 1.73, 95% CI: 1.11–2.69), extracapsular extension (*P* = 0.003, HR: 2.01, 95% CI: 1.26–3.21) and overexpression of UTX (*P* = 0.029, HR: 1.61, 95% CI: 1.05–2.48) were independent prognostic factors of worse OS. The survival outcomes in the univariate and multivariate analyses are shown in Tables 3 and 4.

**Table 3:** Results of univariate and multivariable analyses of prognostic factors for disease-free survival (DFS) in 148 patients with oral tongue squamous cell carcinoma receiving surgical resection

Parameters		Number of patients	Univariate analysis		Multivariable analysis	
			DFS (months)	P value	HR (95% CI)	*P* value
Age						
	<53y/o	70 (47%)	50.2	0.27		
	≧53y/o	78 (53%)	36.7			
Sex						
	male	135 (91%)	38.8	0.15		
	female	13 (9%)	NR			
Pathological T status						
	T1 + T2	90 (61%)	69.8	0.004*		
	T3 + T4	58 (39%)	11.4			
Pathological N status						
	N0	79 (53%)	87.9	< 0.001*		
	N1 + 2 + 3	69 (47%)	13.2			
Pathological 8th AJCC Stage						
	I + II	60 (41%)	NR	0.001*		
	III + IV	88 (59%)	13.4			
Histologic grade						
	1	84 (57%)	57.7	0.08		
	2 + 3	64 (43%)	23.3			
Vascular invasion						
	Absent	124 (84%)	52.6	0.018*		
	Present	24 (16%)	11.0			
Perineural invasion						
	Absent	81 (55%)	77.5	0.008*		
	Present	67 (45%)	14.0			
Extracapsular extension						
	Absent	111 (75%)	64.5	< 0.001*		
	Present	37 (25%)	7.9		2.22 (1.42–3.47)	< 0.001*
Surgical margin						
	Negative	137 (93%)	45.9	0.08		
	Positive	11 (7%)	12.8			
Smoking history						
	Absent	26 (18%)	NR	0.07		
	Present	122 (82%)	33.0			
Alcohol history						
	Absent	30 (20%)	47.3	0.54		
	Present	118 (80%)	33.0			
Betel-nut chewing history						
	Absent	36 (24%)	45.9	0.29		
	Present	112 (76%)	38.8			
UTX expression						
	Overexpression	64 (43%)	20.6	0.006*	1.61 (1.05–2.45)	0.028*
	Low expression	84 (57%)	69.8			

AJCC, American Joint Committee on Cancer. NR, not reach; HR, hazard ratio; CI, confidence interval. *Statistically significant

**Table 4:** Results of univariate and multivariable analyses of prognostic factors for overall survival (OS) in 148 patients with oral tongue squamous cell carcinoma receiving surgical resection

Parameters		Number of patients	Univariate analysis		Multivariable analysis	
			OS (months)	*P* value	HR (95% CI)	*P* value
Age						
	<53y/o	70 (47%)	57.7	0.43		
	≧53y/o	78 (53%)	51.1			
Sex						
	male	135 (91%)	51.1	0.07		
	female	13 (9%)	NR			
Pathological T status						
	T1 + T2	90 (61%)	82.2	< 0.001*		
	T3 + T4	58 (39%)	13.2		1.73 (1.11–2.69)	0.015*
Pathological N status						
	N0	79 (53%)	NR	< 0.001*		
	N1 + 2 + 3	69 (47%)	14.8			
Pathological 8th AJCC Stage						
	I + II	60 (41%)	NR	< 0.001*		
	III + IV	88 (59%)	22.5			
Histologic grade						
	1	84 (57%)	64.5	0.18		
	2 + 3	64 (43%)	33.0			
Vascular invasion						
	Absent	124 (84%)	68.4	0.004*		
	Present	24 (16%)	11.4			
Perineural invasion						
	Absent	81 (55%)	82.2	0.022*		
	Present	67 (45%)	30.7			
Extracapsular extension						
	Absent	111 (75%)	76.5	< 0.001*		
	Present	37 (25%)	11.7		2.01 (1.26–3.21)	0.003*
Surgical margin						
	Negative	137 (93%)	57.6	0.031*		
	Positive	11 (7%)	13.2			
Smoking history						
	Absent	26 (18%)	NR	0.031*		
	Present	122 (82%)	40.3			
Alcohol history						
	Absent	30 (20%)	68.4	0.34		
	Present	118 (80%)	41.2			
Betel-nut chewing history						
	Absent	36 (24%)	NR	0.16		
	Present	112 (76%)	45.5			
UTX expression						
	Overexpression	64 (43%)	23.0	0.006*	1.61 (1.05–2.48)	0.029*
	Low expression	84 (57%)	69.8			

AJCC, American Joint Committee on Cancer. NR, not reach; HR, hazard ratio; CI, confidence interval. *Statistically significant

### Inhibition of UTX by GSK-J4 decrease the abilities of migration and invasion of OTSCC

**Fig. 4:**
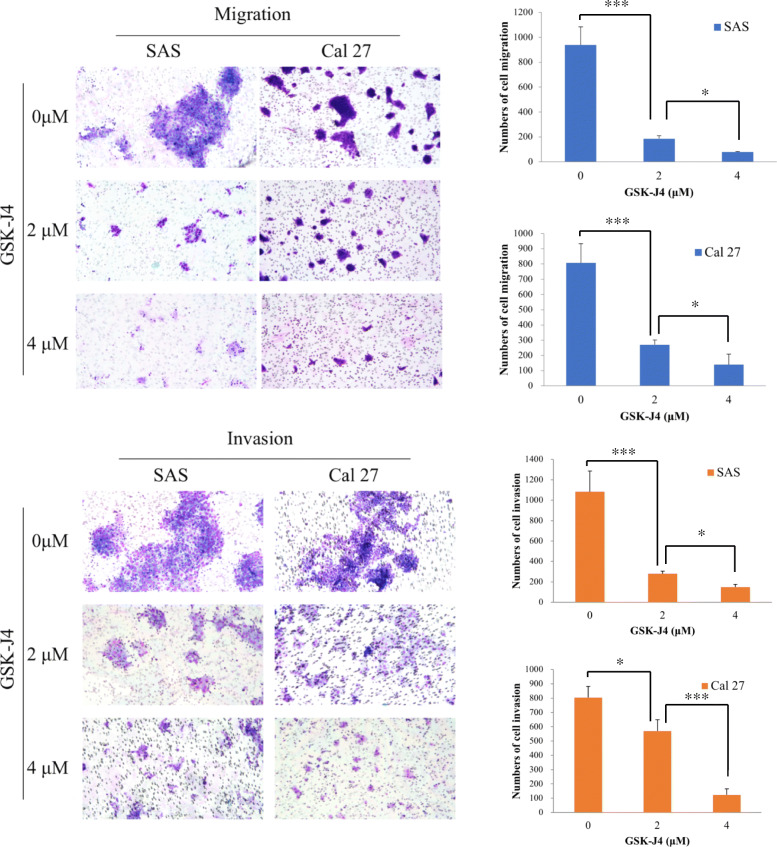
Transwell migration and invasion assays using SAS and Cal 27 cell lines treated with GSK–J4 at different concentrations. Columns, mean; bars, standard deviation. Significant difference: *P < 0.05 and ***P < 0.001

**Fig. 5:**
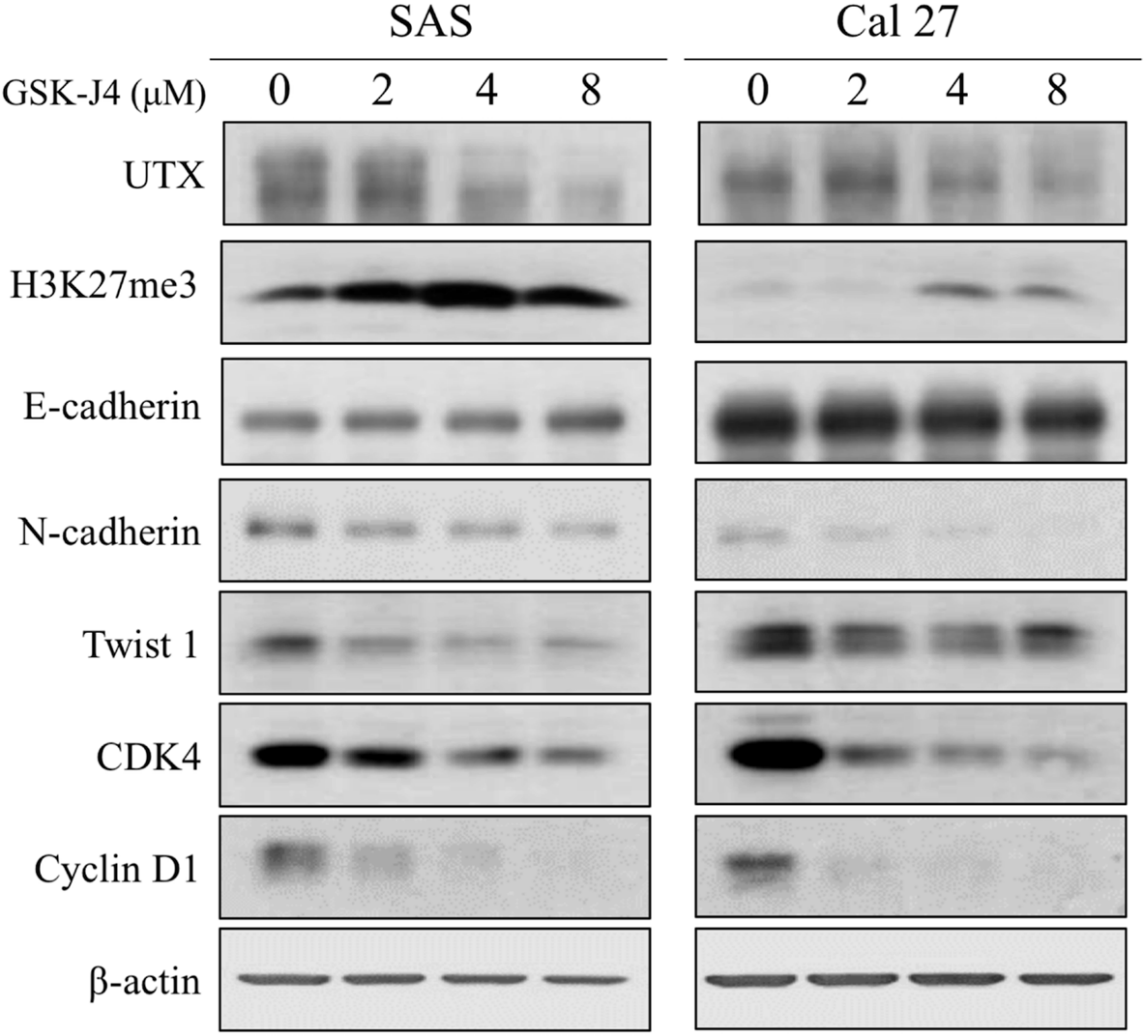
Western blot analysis of UTX expression and the downstream signaling pathway in the SAS and Cal 27 cell lines. The protein expression profiles of UTX, H3K27me3 status, EMT markers, cyclin D1 and CDK4 were examined in the presence or absence of GSK-J4 treatment in the OTSCC cells by Western blotting

Accumulating evidence indicated that GSK-J4, a pharmacologic inhibitor, is able to inhibit UTX activity that acts on specifically H3K27me3 which participates in cancer progression. To analyze the effect of UTX in OTSCC, we determined the cellular motility of OTSCC with GSK-J4 treatment by Transwell assay. Transwell assay results revealed that cells treated with GSK-J4 in a dose-dependent manner significantly reduced the number of invaded and migrated cells, compared to cells without GSK-J4 treatment (Fig. 4). The observation demonstrated that, at least in SAS and Cal27 cell lines, inhibition of UTX by GSK-J4 could suppress the motility of OTSCC. Furthermore, the Western blot analyses were performed to determine the expressions of UTX, H3K27me3 status, EMT (epithelial-mesenchymal transition), and GSK-J4-regulated targets in the OTSCC cell lines upon GSK-J4 treatment. Our data showed that the protein expressions of UTX, N-cadherin, Twist1, CDK4 and cyclin D1 were downregulation, but H3K27me3 status and E-cadherin were upregulation in GSK-J4-treated cell lines compared to that in the control cells (Fig. 5). Collectively, these data demonstrated that UTX expression participates in the motility of OTSCC.

## Discussion

Histone demethylases are epigenetic regulators; they are involved in several cellular processes and in organogenesis. In addition, they are also associated with cancer, and play a crucial role of carcinogenesis, whether acting as oncogenes, or as tumor suppressor genes. H3K27me3 is regulated by the histone demethylase UTX, and results in conversion to H3K27me2 or H3K27me1; this results in a change in chromatin conformation from a repressive form to an active form [23]. UTX has been reported to be involved in the process of cancer, including growth, differentiation, migration, invasion, and apoptosis, through several signaling pathways; however, there is controversy regarding the expression pattern of UTX in different malignancies [13–16, 19].

In our study, the overexpression of UTX and extracapsular extension were independent prognostic factors of worse DFS and OS in both univariate and multivariate analyses. However, the role of UTX acting as an oncoprotein or as a tumor suppressor remains controversial. Growing evidences have shown that decreased H3K27me3 levels may result in tumor cell transcription and EMT, contributing to poor prognosis in some malignancies such as breast cancer, lung cancer and T cell acute lymphoblastic leukemia (T-ALL) [13, 24–27]. In general, there were balance between EZH2 and UTX and its protein interactors within the COMPASS complex, which activates and inhibits H3K27 methylation, respectively. Lower expression of UTX may result in an imbalance status, reduction of inhibiting H3K27 methylation by UTX and activation of H3K27 methylation by EZH2, contributing to tumor cell transcription and poor prognosis [26]. In lung adenocarcinoma, UTX and its COMPASS family member, MLL4, were statistically increased in brain metastasis compared to primary tumor; they also induced the expression of several EMT-transcription factors (TFs), such as ZEB1, Slug and Twist, indicating that UTX may regulate the expression of EMT-TFs epigenetically and affect distant metastasis [25]. In T-ALL, UTX is reported to be associated with TAL1, an oncogenic TF, and is recruited by TAL1 to its target genes. Suppression of UTX in several TAL1-positive cell lines result in an increase in apoptosis, whereas overexpression of UTX enhances tumor cell proliferation [27]. Kim et al. reported that the inhibition of UTX resulted in significant reduction in the proliferation and invasiveness of breast cancer cells in vitro and in a mouse xenograft model [13]. Another study revealed UTX regulates the expression of estrogen receptor α target genes which are associated with development of breast cancer [16]. The loss of UTX leads to a significant reduction in estrogen-induced cell proliferation in a human breast cancer cell line; in contrast, overexpression of UTX promotes cell migration [16]. Therefore, overexpression of UTX may lead to reduce H3K27 methylation, inhibit apoptosis, promote tumor cell transcription and distant metastasis and enhance EMT, contributing to poor prognosis. Our study concluded the overexpression of UTX was found to be associated with worse DFS and OS, indicating the significance of UTX as a tumor oncogene in OTSCC.

There were some studies which reported sex-specific difference in the expression of UTX. Xu et al. showed that higher expression of UTX was found in female mice than in male mice in most brain regions except in the amygdala [28]. This discordance may result from differences in chromatin remodeling on the X and Y chromosomes. In addition, a Belgian study demonstrated that UTX mutations were exclusively present in male T-ALL patients and allelic expression analysis showed UTX escapes X-inactivation in female T-ALL lymphoblasts and normal T cells [29]. In T-ALL, UTX functions as a tumor suppressor and T-ALL driven by UTX inactivation displays collateral sensitivity to pharmacologic H3K27me3 inhibition [29]. In contrast, there were no significant difference of gender distribution in our study, the percentage of female patients in the UTX overexpression and low expression groups were 9 and 8%, respectively (Table 2). Although female patients were found to have longer DFS and OS than male patients in the univariate and multivariate analyses, there was no statistical difference; the better DFS and OS in the female patients may be caused by too lower proportion of female patients (only 9%), resulting in biases existed.

GSK–J4 is a potent dual inhibitor of the H3K27me3 demethylases JMJD3 and UTX, and has been reported to be involved in many physiological and pathological processes. Growing evidence has addressed the effect of this drug in immune cells [30]. GSK–J4 was able to modulate inflammation by affecting dendritic cells, causing an increase in the expression of tolerogenic molecules, and a decrease in the secretion of proinflammatory cytokines [31]. GSK–J4 was also found to inhibit the activity of H3K27 demethylase to suppress T helper 17 cell differentiation as seen in in vitro studies, which suggests that it may be considered as a potential novel therapeutic target for suppressing autoimmune or inflammatory responses [32]. H3K27me3 has been related to the differentiation of normal stem cells and cancer cells, and H3K27 methylation may play a crucial role in inhibiting the maintenance of cancer stem cells [33]. In non-small cell lung cancer, GSK–J4 was able to induce cell death and inhibit the proliferation of tumor cells, irrespective of the genetic mutation status or chemotherapy resistance [34]. Therefore, GSK–J4 may represent a promising anticancer agent.

Our study also had several limitations. First, the small size of the study population may limit this study’s statistical significance. Second, the percentage of female patients were relatively low (only 9%) so it was difficult to avoid selection bias in this retrospectively designed study. Third, the association between UTX and downstream pathways was not fully investigated; additionally, the mechanism of modulation of cancer cell growth and modulation by UTX was not examined. However, to the best of our knowledge, this current study enrolled the largest number of OTSCC patients who underwent surgical resection, and may be helpful to understand the role of UTX in the prognosis of OTSCC.

## Conclusion

Our study suggests that UTX plays an important role in the process of OTSCC, and that the overexpression of UTX is an independent prognostic factor of poor prognosis in OTSCC patients who received surgical resection. Further research with a large population is needed to confirm our findings, and to clarify the complex mechanism of UTX action in OTSCC.

## Supplementary Information

**Additional file 1.** Supplementary Fig. S1. The original data of western blot analyses.

## Data Availability

The datasets used and analyzed during the current study are available from the corresponding author on reasonable request.

## Abbreviations

OTSCC: oral tongue squamous cell carcinoma
UTX: ubiquitously transcribed tetratricopeptide repeat on chromosome X
H3K27: H3 lysine 27
Rb: Retinoblastoma
ECOG: Eastern Cooperative Oncology Group
PS: Performance Status
IRS: immunoreactive score
H3K27me3: tri-methylation of histone H3 lysine 27
DFS: disease-free survival
OS: overall survival
HR: hazard ratio
CI: confidence interval
EMT: epithelial-mesenchymal transition
T-ALL: T cell acute lymphoblastic leukemia
TF: transcription factor
