# Supplementary figures and images for: Overexpression of UTX promotes tumor progression in Oral tongue squamous cell carcinoma patients receiving surgical resection: a case control study
Source: BMC Cancer. 2021 Sep 1;21:979. doi: 10.1186/s12885-021-08726-3 (PMC8408955; doi:10.1186/s12885-021-08726-3)

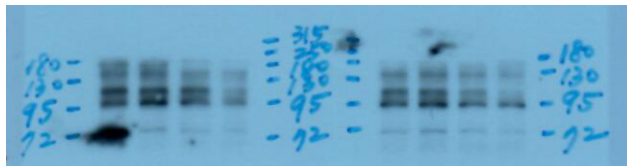

UTX

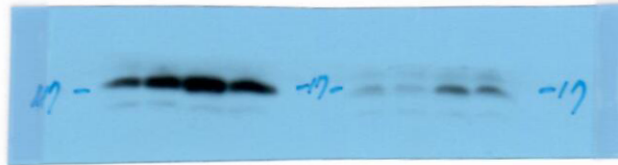

H3K27me3

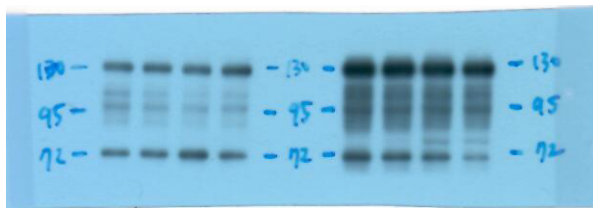

E-cadherin

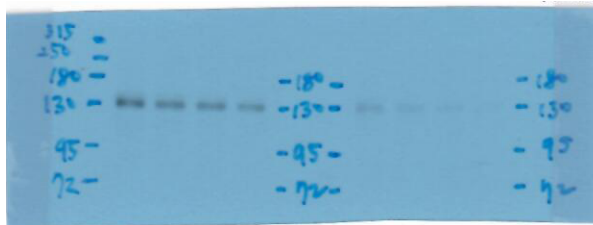

N-cadherin

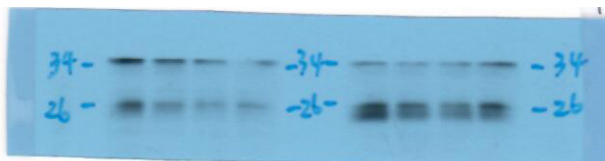

Twist1

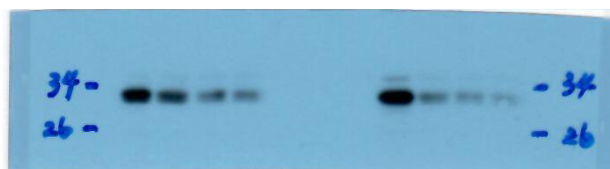

CDK4

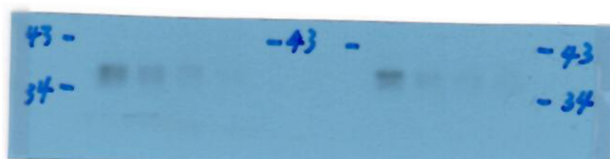

Cyclin D1

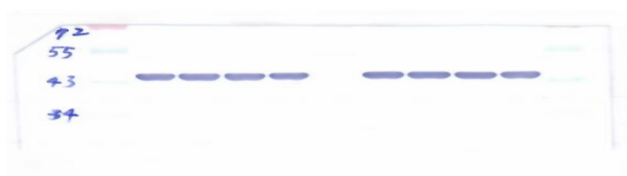

actin

Supplement: Supplementary file 1 — Additional file 1. Supplementary Fig. S1. The original data of western blot analyses. [file 12885_2021_8726_MOESM1_ESM.pdf]
